# Optimization Design and Flexible Detection Method of a Surface Adaptation Wall-Climbing Robot with Multisensor Integration for Petrochemical Tanks

**DOI:** 10.3390/s20226651

**Published:** 2020-11-20

**Authors:** Minglu Zhang, Xuan Zhang, Manhong Li, Jian Cao, Zhexuan Huang

**Affiliations:** School of Mechanical Engineering, Hebei University of Technology, Tianjin 300130, China; zhangml@hebut.edu.cn (M.Z.); 201811201016@stu.hebut.edu.cn (X.Z.); 201921202068@stu.hebut.edu.cn (J.C.); lnzx0508@163.com (Z.H.)

**Keywords:** wall-climbing robot, passive adaptive mechanism, magnetic circuit optimization, flexible detection method

## Abstract

Recently, numerous wall-climbing robots have been developed for petrochemical tank maintenance. However, most of them are difficult to be widely applied due to common problems such as poor adsorption capacity, low facade adaptability, and low detection accuracy. In order to realize automatic precise detection, an innovative wall-climbing robot system was designed. Based on magnetic circuit optimization, a passive adaptive moving mechanism that can adapt to the walls of different curvatures was proposed. In order to improve detection accuracy and efficiency, a flexible detection mechanism combining with a hooke hinge that can realize passive vertical alignment was designed to meet the detection requirements. Through the analysis of mechanical models under different working conditions, a hierarchical control system was established to complete the wall thickness and film thickness detection. The results showed that the robot could move safely and stably on the facade, as well as complete automatic precise detection.

## 1. Introduction

With the rapid development of industries, an increasing number of spherical and cylindrical tanks have been used to store industrial products in the petrochemical field. Different degrees of damage in storage tanks have gradually emerged due to the open environment and natural aging, and regular maintenance has been adopted to ensure the safety of operation. However, traditional maintenance methods require a large number of humans and resources that are inefficient, costly, and dangerous [1,2,3,4,5]. Thus, developing a reliable and flexible wall-climbing robot has become a hot spot in the field of tank maintenance, as such a robot can realize the high precision detection of different detection modules under high risk and in complex petrochemical tanks [6,7,8,9,10].

At present, the adsorptive, moving, and detection mechanisms of wall-climbing robots have been extensively studied. Some typical robot systems have been developed and applied in various fields. The adsorption mechanism is the primary condition to ensure robot movement on a facade. Wall-climbing robots have different adsorption mechanisms for different working surfaces and moving modes. Numerous studies have revealed the following five adsorption modes: permanent magnet, electromagnetic, negative pressure, molecular force, and mixed adsorption [11,12,13,14,15,16,17]. Navaprakash et al. [18] used the principle of negative pressure adsorption to design an adsorption mechanism and verified its safe and stable adsorption on non-magnetic facades through software simulation. Chen et al. [19] designed a wall-climbing robot that uses a rotational-flow suction unit to realize climbing rough walls and overstepping small obstacles. Demirjian et al. [20] designed a caterpillar wall-climbing robot based on bionic principles that uses binder materials and breaks with traditional adsorption concepts. Seriani et al. [21] used wall-climbing robots on both sides of a wall to adsorb each other so as to realize the safe adsorption and stable movement on a non-magnetic wall. Wang et al. [22] optimized the magnetic circuit through the finite element analysis method and designed a new type of permanent magnet wheel with the same magnetic pole array arrangement that considerably improved the adsorption efficiency of the magnet. Wen [23] proposed an adjustable variable magnetic adsorption mechanism to realize the stability detection of a robot on the outer walls of storage tanks. Eto et al. [24] innovatively designed a two degrees-of-freedom (DOF) rotating magnetic attachment mechanism that maintains the optimal adsorption state of the magnet through passive adjustment and realizes safe and stable adsorption on different walls. Xiao et al. [25] designed a new steady-state permanent magnet adsorption operation mechanism to accomplish stable adsorption on complex facades. Fan et al. [26] combined electromagnetic and internal force compensation principles to realize the fast, controllable adsorption and separation of wall-climbing robots.

Many research institutions have developed a large number of wall-climbing robots for industrial applications based on the above adsorption mechanisms by combining mobile mechanisms and detection methods. By integrating viscous materials and a wheel-legged moving mechanism, Amirpasha et al. [27] innovatively proposed a wheeled foot-climbing robot that can achieve large obstacle crossing and wall transition. Wang et al. [28] creatively designed a bipedal, three-DOF wall-climbing robot to realize the detection of wind fan blades. Huang et al. [29] designed a crawler robot for ship detection by integrating a caterpillar structure and the magnetic adsorption mechanism that could realize the large-area detection of complex walls. Zhang et al. [30] designed a wall-climbing de-rusting robot for ship welds based on the visual recognition method of three-line laser structural light. Zhang et al. [31] developed a crawler wall-climbing robot to remove coatings based on high pressure water jet technology. In addition, numerous wall-climbing robots have been developed for petrochemical maintenance and other fields [32,33,34,35,36,37]. Mizota et al. [38] proposed a control method for the compliant motion of a wall-climbing robot based on propelling wave theory to realize stable and flexible movements on a façade by wall-climbing robots. Wu et al. [39] innovatively proposed a coordinated control method based on task trajectory tracking to realize the compliant detection of robots. Zhang [40] used an intelligent perception system to compliantly control a robot and to realize autonomous adaptive full-range detection over complex terrain. Song et al. [41] proposed an intelligent discrete trajectory tracking control algorithm based on the improved Dual-Heuristic Dynamic Programming (DHP) algorithm to solve the circular trajectory movement of a robot on a vertical wall.

Numerous wall-climbing robots have been developed and applied for petrochemical maintenance. However, current research is generally in the bottleneck state due to the limitations of reliable adsorption, surface adaptability, and detection devices, and the following three problems should be urgently solved. (1) Permanent magnet adsorption mechanisms have low magnetic energy utilization and adsorption capacity due to the limited transfer mechanism analysis of the multimedium magnetic circuit. (2) Moving the existing wall-climbing robots smoothly on curved surfaces with changeable morphologies is difficult due to insufficient studies on the passive flexible adaptive moving mechanism. (3) Achieving the vertical alignment of a probe for different detection modules while sticking to the facade is difficult for existing detection mechanisms, thus affecting detection effects and accuracy

Here, a wall-climbing detection robot that can realize multimode non-destructive testing on different walls is proposed on the basis of the above-mentioned problems. A high performance permanent magnet wheel was designed on the basis of magnetic circuit optimization to solve the safety adsorption problem, and the rapid demagnetization structure of the wheel was designed to facilitate the robot’s removal from the wall after detection. Different from the traditional wall climbing mechanism with rigid connection, the wheels in this paper were flexibly connected with the moving mechanism to form a pseudopodia robot that could adapt to curved surfaces and move flexibly on the surfaces of spherical and cylindrical storage tanks. In order to improve the detection accuracy and efficiency of existing testing equipment, a flexible adaptive detection mechanism with multi-DOFs is proposed to passively adapt to wall surfaces by integrating a hooke hinge mechanism. A dynamic model of the wall-climbing robot was established on the basis of different working conditions to solve the momentum distribution problem of wheels under different motion modes. Through different process controls, the robot can use ultrasonic and eddy current probes to detect the thicknesses of wall and paint film, respectively. Experiments were conducted on a 5-mm-thick cylindrical tank surface to test the structure and detection capability of the robot. The experiments showed that the robot can move flexibly and stably on different facades. Simultaneously, the robot can accurately detect the thicknesses of walls and paint films by carrying different detection probes that can replace manual work to a certain extent.

The remainder of this paper is organized as follows. Section 2 introduces the structure of the detection robot, which mainly includes the magnetic adsorption moving mechanism and the passive flexible detection mechanism. Section 3 establishes mechanical analysis models for different working conditions and motion modes to determine the minimum adsorption and driving forces of safe and stable motions. Section 4 introduces the hardware composition of the control system and flexible detection process control flow with multiple detection capabilities. Section 5 presents the experimental process and analysis results. Section 6 provides several conclusions drawn from this research.

## 2. Introduction to Detection Robot

**Figure 1:**
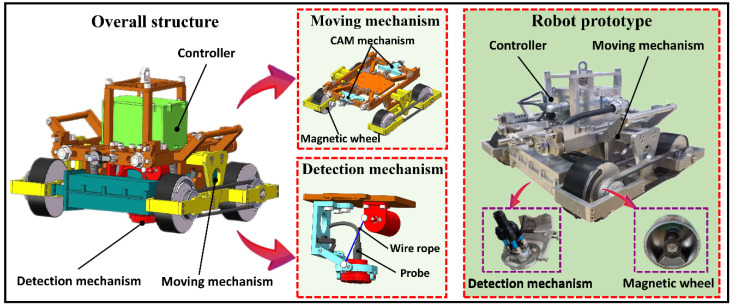
Overall structure of the wall-climbing detection robot.

A wall-climbing detection robot adapted to different curvature walls was developed while considering the varied morphology of petrochemical tanks. The robot mainly comprised an adaptive moving mechanism, magnetic adsorption wheels, and a flexible detection mechanism with multi-DOF. The working environment of the robot comprises facades with different curvatures. Thus, solving the problems of the safe adsorption and stable movement of the moving mechanism, as well as the flexible adaptation and accurate measurement of the detection mechanism, was necessary. Therefore, a wall-climbing robot with flexible detection was developed. This robot can steadily adsorb and complete different detection tasks on different facades to meet the requirements of petrochemical tank detection. A high performance magnetic wheel structure that can be quickly demagnetized is also proposed. This structure coordinates the design of the multi-DOF moving mechanism to passively adapt to different curvature walls to ensure safe and stable movement. A flexible detection mechanism was designed in accordance with the operational requirements of the different detection modules by integrating rope pulling and a hooke hinge mechanism to realize the self-adaptive vertical alignment of the probe to adapt to different detection techniques. The detection robot can realize the precise movement and action of different detection process flows through a state control strategy and finally complete the wall detection tasks. The specific structure of the robot is shown in Figure 1.

### 2.1. Magnetic Wheel

#### 2.1.1. Structural Design of the Magnetic Wheel

**Figure 2:**
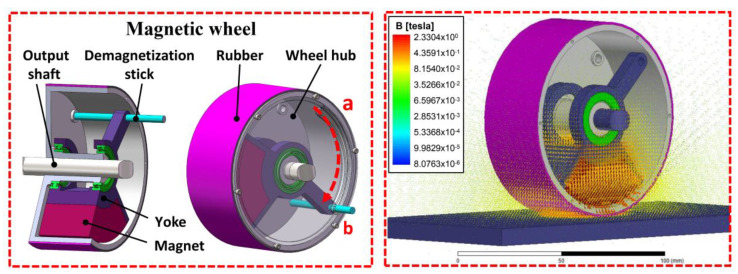
Magnetic wheel structure with fast demagnetization.

The permanent magnet adsorption mechanism is the crucial point in the design of a wall-climbing robot, because it is directly related to the safe absorption and stable movement on a wall surface. The robot movement is stable and the safety factor is large when the magnetic wheel adsorption capability is strong. However, the friction between magnetic wheels and the wall surface increases with the adsorption force and the resistance to be overcome in the movement is large, thus leading to a high driving torque. Simultaneously, detachment from the wall becomes difficult for the magnetic wheel after completing an avoidance detection task. Therefore, designing a lightweight wheel with strong adsorption was the pivotal technical problem to be solved in this paper. A new method using the combination of fan-shaped permanent magnet and yoke iron as the excitation source is proposed to improve the utilization rate of the magnet. At the same time, in order to detach the robot from the wall after completing the task, a fast demagnetization method was designed by using the lever principle. The specific structure is shown in Figure 2.

In the process of the adsorption force production of the magnetic wheel, most magnetic sensing lines come from a small part of the magnet close to the wall surface. Therefore, a radial magnetized fan magnet (Nd2Fe14B) was selected as the excitation source to reduce the weight and provide a strong adsorption force. Yoke iron was used to collect magnetic induction lines because of its high permeability that can reduce magnetic flux leakage and improve the utilization ratio of magnetic energy. Figure 2 shows that the fan magnet was placed in the suspension of the wheel, which could rotate relative to the wheel hub. When the output shaft transmits motion to the hub through a key, the permanent magnet always remains relatively still with the wall and does not rotate with the hub, which not only maintains a constant adsorption force but also avoids relative motion with the wheel. Actively reducing the adsorption force between the magnetic wheel and the wall, that is, the magnetic wheel demagnetization, is necessary to facilitate the robot detachment from the wall after the detection task. A small tangential force can be used in the adsorption state to force magnet rotation relative to each other, which can reduce the adsorption force between the magnet and the wall. Thus, a fast demagnetization mechanism was designed on the basis of the lever principle, which could facilitate magnet rotation around an output axis, thus completing the demagnetization.

#### 2.1.2. Optimization of Magnetic Wheel

The magnetic wheel structure was optimized to obtain a high performance and lightweight magnetic wheel. The adsorption force of a magnetic wheel whose outside diameter and width are fixed is affected by the air gap h, the thickness of yoke iron H, and the shape of the magnet (the inner radius $R_{in}$ and angle of the magnet θ). The electromagnetic field analysis software Ansoft was used to analyze the magnetic field strength of the permanent magnet and to determine the relationship between magnetic wheel parameters and the magnetic field strength to realize the lightweight of the magnetic wheel and ensure the reliability of adsorption. This analysis provided a reference for motion assessment and improved the magnetic utilization rate.

**Figure 3:**
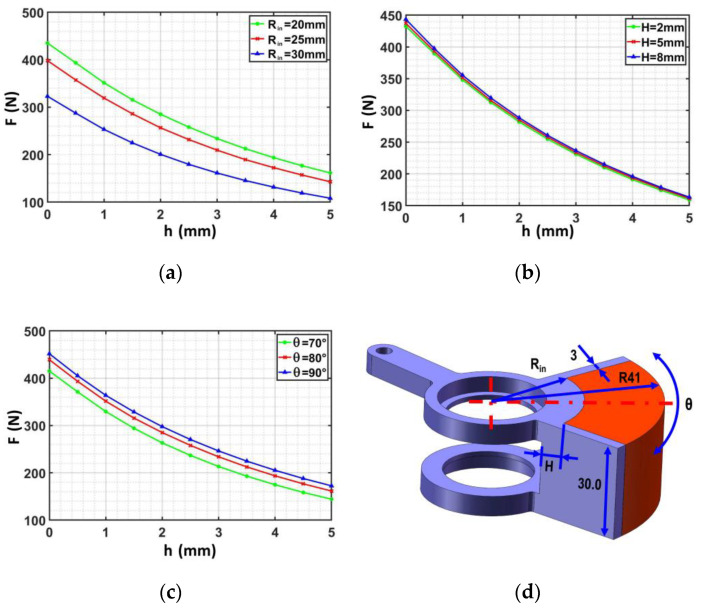
Influence of magnetic wheel parameters on the adsorption force: (**a**) The relationship between the adsorption force and the air gap height under different inner radius of the magnet, (**b**) the relationship between the adsorption force and the air gap height under different yoke iron thicknesses, (**c**) the relationship between the adsorption force and the air gap height under different magnet center angles, and (**d**) description of magnetic wheel structure and size.

According to the principle of a single variable, the relationship between wheel adsorption force F and variable can be obtained by changing the air gap h, inner radius $R_{in}$, and angle of magnet θ. The simulation results are shown in Figure 3.

The magnetic wheel adsorption force was found to be inversely proportional to the distance from the wall according to the information in the three above-mentioned figures; the adsorption force further away from the was found to be worse. Figure 3a shows that an increase in the inner radius $R_{in}$ could lead to a contained high magnetic energy, a high magnetic field intensity that could be excited, and a strong adsorption force. Figure 3b indicates that the capability of the yoke iron to collect magnetic induction lines was found to increase with the yoke iron height H. This phenomenon complicates the magnetic saturation production and enhances the utilization ratio of magnetic energy products to improve magnetic field strength and adsorption force. Figure 3c reveals that the effective transfer area between the magnet and the wall surface was found to increase with the angle of the magnet θ, which improves the adsorption performance of the magnet. Considering the volume limitation of the wheel, the shape of the magnet was optimized in accordance with the functional relationship between the magnetic field strength and the geometric parameters of the magnetic wheel (h, $R_{in}$, θ, and H). Continuous nonlinear programming has the capability of using the response surface method to approximate the finite element response characteristics, which is very suitable for solving the optimization problem of finite variables. Therefore, the continuous nonlinear programming in Ansoft Maxwell was adopted to optimize the three parameters of the magnetic wheel. The iterative process is complex and tedious but is commonly used; thus, comprehensively describing the solution process is unnecessary. Finally, a permanent magnet wheel with good performance was designed, and the specific mechanism size is shown in Table 1.

The magnetic wheel adsorption experiment was conducted on an arc facade to test the adsorption capability of the magnetic wheel. The adsorption capacity of the wheel was tested in horizontal, vertical, and oblique states. The actual adsorption force was obtained by reading the maximum pull value of the magnetic wheel in the adsorption state on the wall through the dynamometer (the pull value of the wheel when it leaves the wall is the instantaneous maximum pull value). The influence of gravity in all cases was removed in the data recording process. The specific values are shown in Table 2.

The actual adsorption force of the magnetic wheel could be obtained as 120 N by averaging the above values. The adsorption capacity can meet the requirement of safety adsorption of wall-climbing robot.

Thus far, a wheeled adsorption mechanism was innovatively designed. A lightweight permanent magnet wheel with strong adsorption capability was obtained through magnetic circuit optimization design and multivariable simulation optimization. The actual adsorption capacity of the magnetic wheel under different working conditions was then measured by experiments, and the adsorption performance of the magnetic wheel was verified.

### 2.2. Passive Adaptive Moving Mechanism

**Figure 4:**
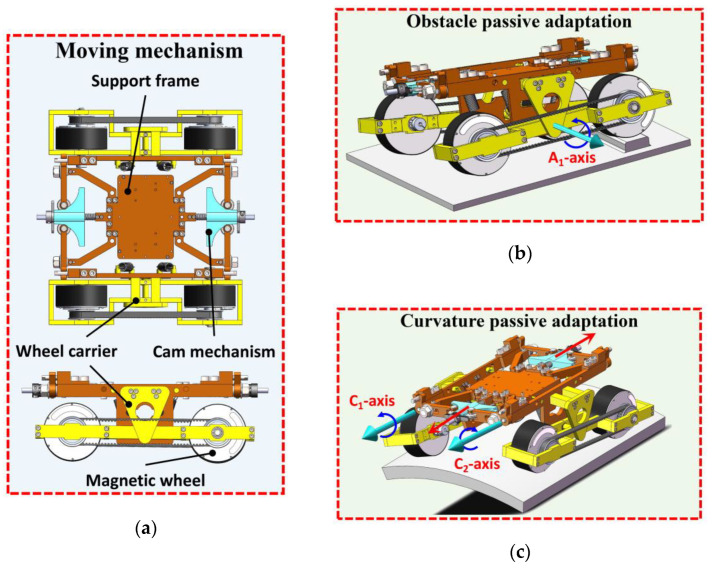
Pseudopodia flexible moving mechanism: (**a**) Moving mechanism structure, (**b**) obstacle crossing process, and (**c**) surface adaptation process.

A detection robot works on a circular or spherical facade, and the adaptability of the moving mechanism to the complex wall is directly related to the movement safety and stability. Achieving the adaptability of small curvature tanks is difficult for traditional moving mechanisms, which will easily lead to slipping and instability, thus affecting work efficiency and operation safety. Moreover, realizing the stable movement of a robot on a wall becomes a problem. The possible instability of the detection robot was analyzed to solve this problem, which mainly includes the following two points. (1) A single front wheel is forced to leave a wall surface when a detection robot encounters an obstacle. Another wheel on the same side with a similar connection also leaves the surface due to the rigidity of the robot. This phenomenon directly leads to a sharp decline in the adsorptive capacity of the robot on the wall surface, thus making the robot prone to instability. (2) A robot’s movement on the curved surface leads to an incomplete fitting of the angle between wheels and the wall. Ensuring enough adsorption force is difficult, and the decrease in contact area easily causes instability. Different from the traditional wheeled moving mechanism, we combined multi-DOF deformation concept to design an innovative moving mechanism with the ability for surface passive adaptation. The close contact between wheels and the wall was realized by passively adapting the fuselage component, thus ensuring the safe operation of the moving mechanism. The specific mechanism is shown in Figure 4.

A passive adaptive moving mechanism was designed in this paper to improve the adaptability of robots to facades and ensure their safe and stable movement. Figure 4 shows that the moving mechanism comprises the wheel frame, support frame, and cam mechanism. The wheels on the left and right sides are connected with the hand frame through the axes $A_{1}$ and $A_{2}$, respectively, and can rotate about the axes. The cam mechanism is fixed on the front and rear sides of the support frame by springs. The elastic deformation of the spring pushes the cam to move, which drives the wheel frame rotation around the axes $C_{1}$ and $C_{2}$; thus, both wheels fit vertically to the wall. Hence, the robot can be safely adsorbed on different curvature walls. The driving motors adopt diagonal arrangement and transfer power by using a synchronous belt to ensure the driving torque and simplify the control. Figure 4b shows that the right wheel frame rotates around axis $A_{1}$ when the unilateral wheel of the robot encounters obstacles to ensure that each wheel can be reliably adsorbed on the wall surface. This phenomenon avoids the first instability situation. Figure 4c shows that the cam mechanism is passively adjusted to drive the wheel frames on both sides moving to rotate around the axes $C_{1}$ and $C_{2}$ when the robot operates on the circular arc wall. Therefore, the magnetic wheel can closely contact the wall surface, which ensures stable and safe movements. Through the design of the above structure, the wheels on both sides of the robot can be flexibly adjusted with multi-DOF to ensure that each wheel can contact closely to different curvature walls and meet safety adsorption requirements.

### 2.3. Detection Mechanism

**Figure 5:**
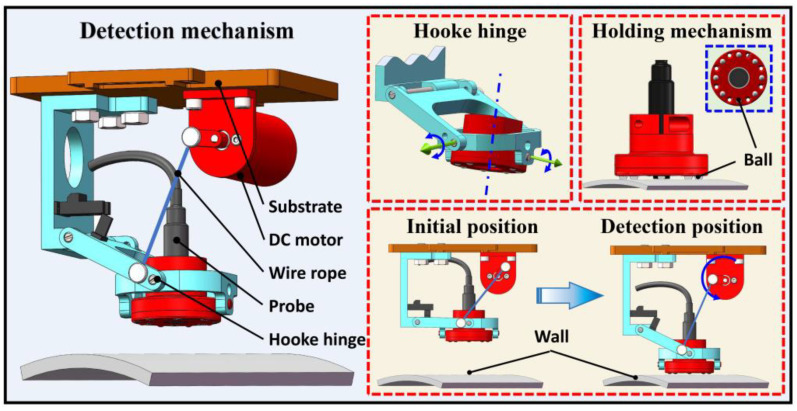
Flexible detection mechanism.

Nondestructive testing has always been highly recommended in the detection methods of petrochemical storage tanks. Ultrasonic and eddy current sensors are needed during the maintenance of petrochemical storage tanks to complete the thickness measurements of the wall and paint film. Different detection tasks require different technological processes, and the relative position between the detection device and the wall surface directly affects the detection effect and accuracy. Therefore, keeping the probe vertically aligned and close to the wall surface is necessary, while active and accurate real-time control increases the difficulty of control. Different from a traditional rigid detection mechanism, an underactuated passive adaptive detection mechanism was designed by integrating a hooke hinge mechanism to meet the precise detection requirements of different walls. The vertical alignment of probes is realized by the passive adaptation of the hooke hinge mechanism, and the probe is pressed tightly to the wall surface by the spring to meet the detection requirements. The specific architecture is shown in Figure 5.

Figure 5 shows that the detection mechanism is fixed on the robot through the substrate. Hooke hinge structures enable a detection mechanism to have three DOFs, which can help detection mechanisms be perpendicular to kinds of complex wall surfaces. The torsion spring is installed on the rotary shaft of the hooke hinge mechanism. This hinge can provide torque force to press the probe on the wall surface to ensure the detection effect and improve detection accuracy. The hooke hinge mechanism is connected with the DC motor rocker arm through a wire rope. The DC motor rotates to lower the probe in the detection state. On the contrary, the DC motor rotates in reverse to lift the probe away from the wall in the non-detection state. The detection mechanism can be manually fixed into an L-shape by the locating pin after the removal of the wall-climbing robot from the wall surface. The holding mechanism in the hooke hinge mechanism is used to fix the detection probe, and different detection modules can be conveniently replaced to complete different detection tasks. In addition, eight stainless steel beads are installed uniformly on the probe holding mechanism to convert sliding friction into rolling friction to avoid damage to the detection wall. The above structure ensures close contact between the end of the detection mechanism and the surface. Therefore, detection efficiency can be guaranteed.

## 3. Mechanical Analysis

The weight and adsorption force of a robot directly affect the safety and movement flexibility when it runs on different facades. A robot must meet the safety requirements of different working conditions and movement modes in the process of continuous detection to realize the full domain detection of petrochemical storage tanks. Here, the critical failure states of the designed robot were analyzed through a mechanical model under different working conditions to obtain the minimum adsorption force of the magnetic wheel and ensure the safe and stable movement of the wall-climbing robot on a facade. Dynamic models were also established for different motion modes, and the robot and each wheel were analyzed to achieve the optimal momentum distribution and optimize the motion performance.

### 3.1. Statics Analysis

**Figure 6:**
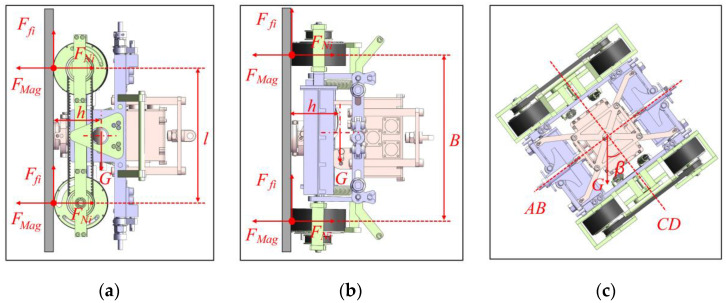
Static model of robot: (**a**) Vertical state, (**b**) horizontal state, and (**c**) oblique state.

In the process of facade movement, a wall-climbing robot is prone to dangerous states, such as static sliding, vertical overturning, horizontal overturning, and oblique overturning. These states affect movement safety and flexibility. Thus, mechanics analysis on a robot must be conducted to determine the minimum adsorption force to ensure safe and stable movement. Here, a mechanical model was established for mechanics analysis, as shown in Figure 6.

Force and moment balance equations were established for the above states based on classical mechanics theory. In order to simplify the calculation process, we proposed the concept of safety factor to compensate for relatively small disadvantages such as cable weight and severe environment. The following static model of the robot was obtained.

(1) $$\{\begin{array}{c} \sum_{i=1}^{4}F_{f}{}_{i}=sG\\ \sum_{i=1}^{4}F_{N}{}_{i}=4F_{Mag}\\ \sum_{i=1,3}(F_{Ni}-F_{Mag})l+sGh_{c}=0\\ \sum_{i=1}^{2}(F_{Ni}-F_{Mag})B+sGh_{c}=0 \end{array}.$$

The meanings of the letters in the formula are shown in Table 3:

The critical condition for the robot to be in a safe and stable state is that all magnetic wheels are on the wall surface, that is, constraints of support force and friction are present and the maximum static friction should be larger than the gravity component. Therefore, the value range of magnetic wheel adsorption force can be obtained as follows: $F_{Mag}\geq sG/4\mu$.

Figure 6c shows that when the robot is inclined to adsorb on the wall surface, the robot may flip around the AB or CD axes in this state. Gravity (G) can be decomposed into Gsinβ and Gcosβ along the direction of AB and CD. Gsinβ and Gcosβ were found to be less than G. Therefore, the calculated critical value of the safety adsorption force is less than the threshold of adsorption force when the robot is vertical and horizontal, as calculated above.

Therefore, the minimum adsorption force required by the robot was obtained to maintain static stability.

### 3.2. Dynamics Analysis

Dynamic analysis was conducted to obtain the optimal driving torque of each motor for the stable movement of the robot in different motion modes. The analysis of various motion modes revealed that the driving torque of other operation modes is less than or equal to that required for vertical upward straight or turning motion. Therefore, the dynamics analysis model of the robot was established in the two situations, and the best driving torque was obtained.

#### 3.2.1. Dynamic Analysis in the Vertical Upward Movement

**Figure 7:**
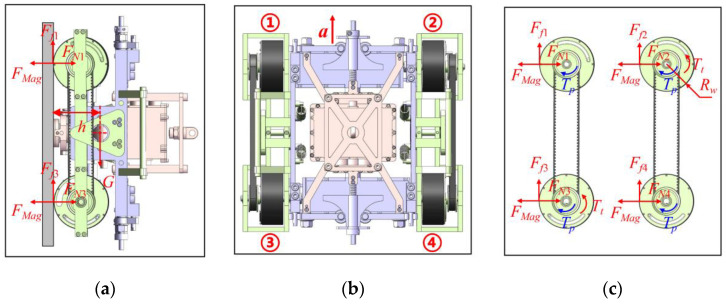
Dynamics model of the robot: (**a**) Side view of the vertical upward state, (**b**) main view of the vertical upward state, and (**c**) force analysis diagram of each wheel.

Dynamic analysis is similar to static analysis when running vertically upward. However, the existence of acceleration and the difference in the friction coefficient should be considered. Assuming that each wheel performs pure rolling motion without sliding, the mechanical model was obtained, as shown in Figure 7.

Rolling resistance was found to be generated due to the deformation of the rubber layer of the wheel, and its deformation was found to be small. Therefore, the moment of rolling resistance compared with other torsional moments could be ignored. The rolling resistance compared with other forces could also be disregarded due to the small rolling resistance coefficient. In order to enable the robot to overcome gravity and move stably, Formula (2) was established according to the principle of force balance, and then the required motor torque was solved.

(2) $$2\frac{T_{t}}{R_{w}}-sG=\frac{sG}{g}a.$$

By simple derivation of Formulas (1) and (2), the required torque of the motor was calculated as follows:

(3) $$T_{t}\geq (\frac{1}{2}+\frac{a}{2g})sGR_{w}.$$

The meaning of the letters in the formula is shown in the following Table 4:

#### 3.2.2. Dynamic Analysis in Steering

**Figure 8:**
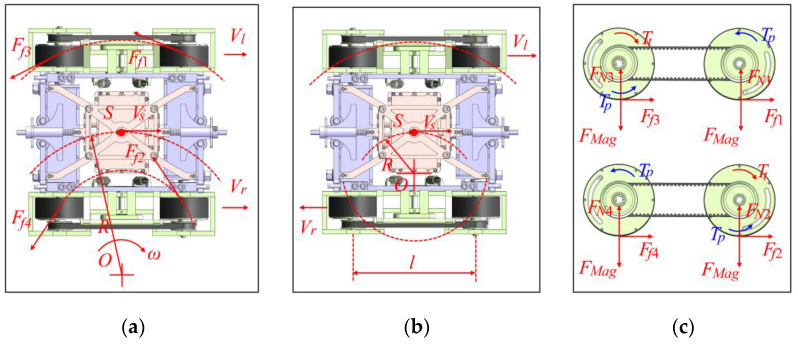
Dynamic analysis in steering: (**a**) Large radius turning state, (**b**) small radius turning state, and (**c**) dynamic analysis of each wheel.

In this research, the wall-climbing robot as found to be able to achieve steering via different speeds of the wheels on each side. The angular speed and steering radius were, respectively, determined by the speed and direction of the wheels on both sides, as shown in Figure 8.

Figure 8 shows the relationship between the rotation speed of wheels on both sides and the turning radius:

(4) $$\{\begin{array}{c} \omega =\frac{V_{l}}{(R+B/2)}=\frac{V_{r}}{(R-B/2)}\\ \alpha =\dot{\omega } \end{array}.$$

The turning radius formula of the robot could be easily obtained according to the speed of the wheels on both side:

(5) $$R=\frac{V_{l}+V_{r}}{V_{l}-V_{r}}\cdot \frac{B}{2}.$$

When R>0.5B, the center of rotation is outside the robot (as shown in Figure 8a); when R<0.5B, the center of rotation is inside the robot (as shown in Figure 8b). The condition of R>0.5B was taken as an example for force analysis. The horizontal to the vertical rotation of the wall-climbing robot was taken as the model to analyze the strained condition. The torque balance formula with point O as the center of rotation, as shown in Formula (6), was established to solve the required output torque of the motor. A mechanical model of the following turning states was obtained:

(6) $$\frac{T_{t}}{R_{w}}(R+\frac{B}{2})+\frac{T_{t}}{R_{w}}(R-\frac{B}{2})-\sum_{i=1}^{4}F_{fi}\frac{l}{2}=J\alpha .$$

In combination with Formulas (4)–(6), Formula (7) could be obtained:

(7) $$T_{t}\geq \frac{J\alpha +2\mu F_{Mag}R_{w}l}{2R}.$$

The dynamics of the two motion modes of the robot, vertical upward motion and turning motion, were analyzed, and the equations were solved to find the motor torque range suitable for the stable operation of the robot.

## 4. Control System

**Figure 9:**
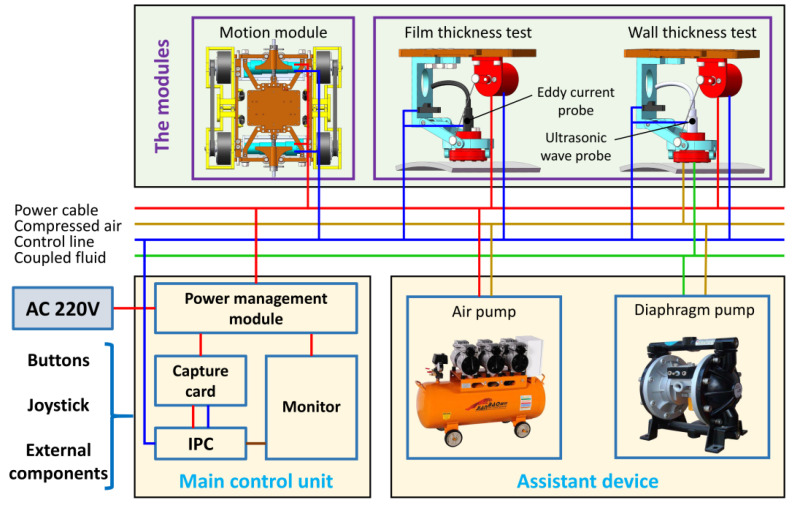
Hardware composition of the control system.

This chapter introduces the hierarchical control system built by an industrial personal computer (IPC) as the upper computer, which uses IPC to realize the planning of the whole detection process and completes the detection task through the hierarchical control of each functional module. The control system mainly includes the precise motion control system of the flexible moving mechanism and the active adjustment control system of the flexible detection mechanism. Limited by the severe environment, wired control is used for remote controls to ensure the stability and accuracy of interactive information transmission. In addition to the basic function of flexible movement on the wall, the detection robot also needs to perform different detection modules for different detections. The robot can measure the thicknesses of the wall and paint film by, respectively, using ultrasonic and the eddy current probes. RS485 communication is adopted to complete the high-speed transmission of real-time detection information to realize multimodule and multimode coordinated detection, and the detection workflow is realized by using a distributed control system to monitor the close cooperation between the components. The specific control system structure is in Figure 9.

The robot is controlled remotely by external input devices, such as buttons in the control box. Control instructions are transmitted to the motors on both sides of the moving mechanism through the RS485. The motion pattern of the robot can be changed by adjusting the rotation speed and direction of the wheels on both sides. Simultaneously, inertial navigation information is used to adjust the running state of the motor to facilitate accurate movement. The distance between the detection device and the wall surface can be actively adjusted by controlling the motor steering in the flexible detection mechanism. The passive adaptability of the robot is used to ensure that the probe is perpendicular to the wall. Data obtained by probes are transmitted back to the main control unit in real-time via RS485 and displayed digitally. The robot can measure the thicknesses of the wall and paint film by, respectively, using ultrasonic and the eddy current probes. An air compressor and a diaphragm pump may be required during wall thickness measurement when using ultrasonic sensors to spray the coupling fluid near the probe to assist the robot in completing the wall thickness detection.

**Figure 10:**
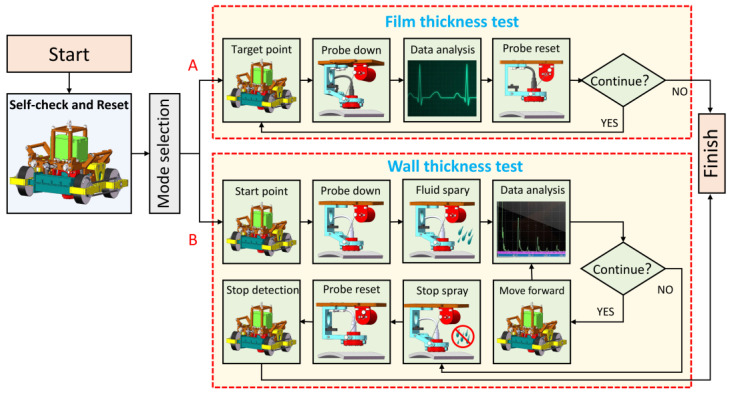
Robot detection workflow. (**A**) in the figure represents film thickness detection. (**B**) in the figure represents wall thickness detection.

The control system of a wall-climbing robot is the key to motion and detection. The control can be divided into three parts: initialization, movement control, and detection control. The motion control realizes the flexible movement of the robot on the wall based on the feedback of the inertial sensor and the active remote control of the user. The detection control is allocated in accordance with different detection modules, which can be called for specific requirements. For example, measuring the position of marking points is necessary when the robot conducts the eddy current detection of paint film thickness while continuous monitoring and the coordination of coupling liquid when the robot conducts ultrasonic detection to wall thickness. The specific control flow chart is shown in Figure 10.

The detection process can be analyzed as follows from the flow in Figure 10.

1. The robot is powered on to perform self-check and reset.
2. The user selects the detection mode:
  A: Film thickness detection.
  B: Wall thickness detection.
3. Different detection modes enter different detection processes:
  A: Film thickness detection:
    (1) The motors are driven to help the robot reach the initial detection point.
    (2) After the robot reaches the predetermined position, the DC motor in the flexible detection mechanism rotates forward to lower the detection probe. The probe cooperates with its passive adaptation mechanism to realize the vertical alignment of the probe.
    (3) After the sensor in the flexible detection mechanism confirms the detection position, the probe collects the paint film thickness information and transmits it back to the main control unit.
    (4) The DC motor reverses to lift the probe, and the robot completes the current position detection.
    (5) The main control unit checks the presence of a termination signal: if no termination signal is present, then the robot moves to the next detection point and repeat steps 2–5; if the termination signal is obtained, then the detection task is stopped.
  B: Wall thickness detection:
    (1) Motors re driven to help the robot reach the initial detection point.
    (2) After the robot reaches the predetermined position, the DC motor in the flexible detection mechanism rotates forward to lower the detection probe. The probe cooperates with its passive adaptation mechanism to realize the vertical alignment of the probe.
    (3) The diaphragm pump sprays coupling fluid on the detection area to assist the detection task.
    (4) The probe collects wall thickness information and returns it to the main control unit.
    (5) The main control unit checks the presence of a termination signal: if no termination signal is present, then the robot continues to run and repeat steps 3–5; if a termination signal is obtained, then the diaphragm pump stops spraying coupling fluid and the DC motor reverses to lift the probe to stop the detection task.
4. The robot completes the detection task and resets.

## 5. Experiment

The authors of this paper designed a flexible and adaptive wall-climbing robot for film and wall thickness detection on curved wall surfaces by combining the magnetic wheel, flexible moving mechanism, and multi-DOF detection unit mentioned above. The key technical parameters of the robot are shown in Table 5.

**Figure 11:**
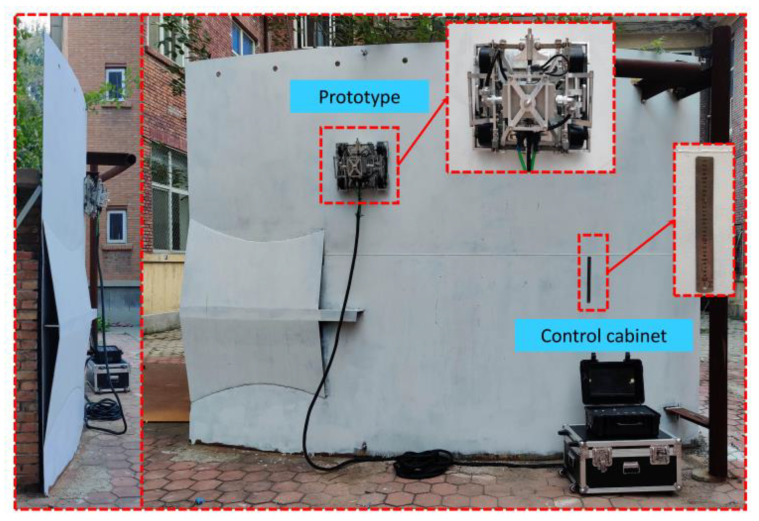
Experimental scene.

This chapter discusses the movement and detection performance tests of the wall-climbing robot to verify the rationality and feasibility of the above-mentioned structure, control system, and the correctness of the mechanical theoretical analysis. The experimental facility mainly comprised the robot system and a cylindrical façade. Figure 11 shows a vertical circular steel plate, with a radius of 8 m and a wall thickness of 5 mm, which was used to simulate the tank environment. The detection robot system included the robot body, the control box, and auxiliary equipment. The air and diaphragm pumps of the auxiliary device provided coupling fluid for ultrasonic thickness detection. Operators controlled the movement and detection of the robot through the control cabinet to complete the detection task of the wall surface.

### 5.1. Movement Performance Test

**Figure 12:**
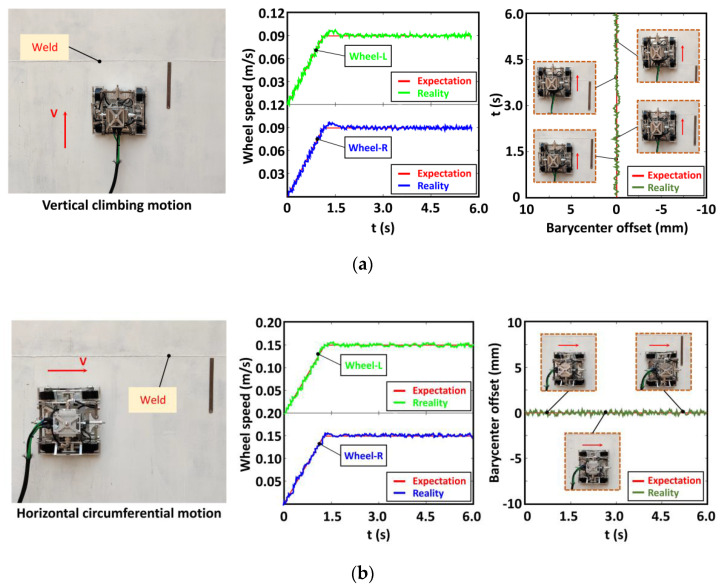
Experimental of wall motion: (**a**) Vertical upward climbing motion test and (**b**) horizontal circumferential motion test.

Experiments on the vertical arc steel plate were conducted to test the stable adsorption and flexible precise movement capability of the robot. The performance of the robot was analyzed by monitoring the rotation speed of each wheel and the change in the position of the robot’s center of mass during its vertical upward and horizontal circumferential movement on the arc plate. Among them, the wheel speed information was obtained by detecting the encoder information of the wheel motor, and the position information of the robot was obtained by the inertial navigation module mounted on the moving mechanism. The details are shown in Figure 12.

The robot could move safely and stably without slipping, falling, and other instabilities during the experiment. This finding indicated that the robot has good adsorption performance and adaptive capability. The synchronous belt is used to drive wheels on the same side. Therefore, the four-wheel robot could be simplified to the form of two wheels on right and left. The data in Figure 12a reveal that the rotation speed of the wheels on both sides during the vertical upward movement remained at 0.09 m/s despite slight fluctuations, and the position of the mass center did not deviate significantly in the horizontal direction. The data in Figure 12b show that the rotation speed of the wheels on both sides remained at 0.15 m/s in the horizontal circular motion of the robot, and the center of mass did not deviate significantly in the vertical direction. The above experimental data prove the steady and accurate robot movement on the circular steel plate.

**Figure 13:**
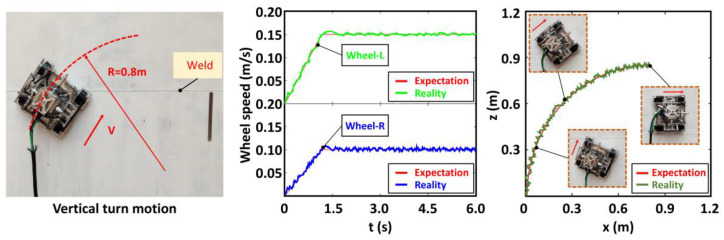
Turn motion test from vertical to horizontal.

A turn right movement experiment was conducted on the vertical circular arc wall to verify the movement flexibility. The robot was controlled to move from vertically upward to horizontally to the right, and the wheel rotation speed and the change of the mass center were detected in this process. The steel plate used in the experiment was expanded along the perimeter to intuitively understand the motion state, and the change curve of the mass center was drawn. The details are shown in Figure 13.

The robot did not lose its stability and completed the right turning movement from the vertical direction to the horizontal direction in the above experiment. The left and right wheel rotation speeds were, respectively, set at 0.15 m/s and 0.09 m/s; therefore, the expected theoretical turning radius was 0.8 m. The figure above reveals that the rotation speed of the wheels on both sides slightly fluctuated up and down around the theoretical value. The adjusted trajectories show that the robot completed the turn, albeit with some deviation due to gravity.

### 5.2. Detection Accuracy Test

**Figure 14:**
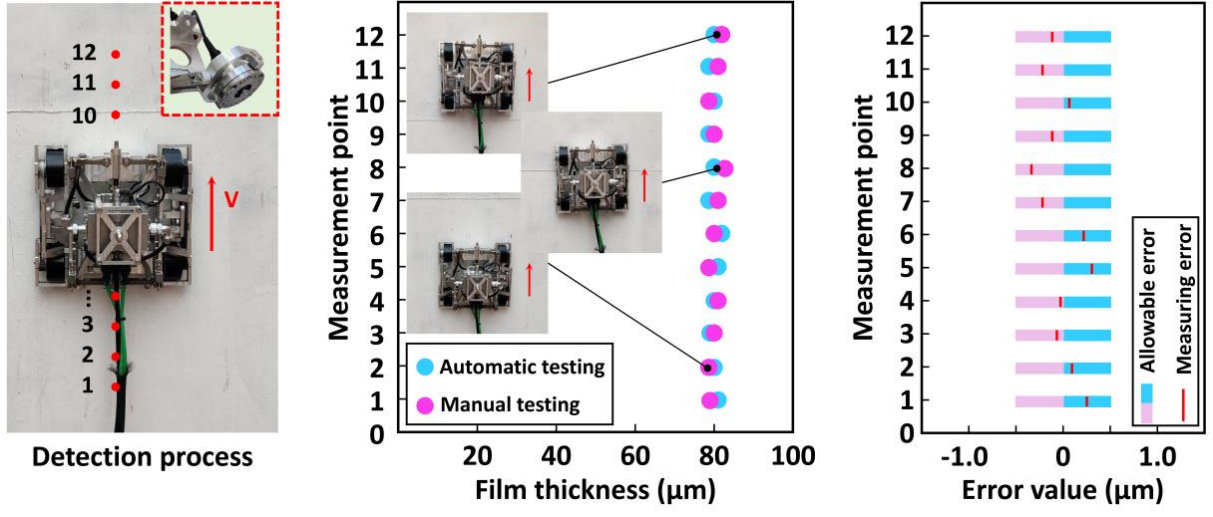
Measurement experiment of film thickness.

The wall-climbing detection robot could carry different detection equipment to detect a wall surface. Measurements of the paint film and wall thicknesses were taken as examples to conduct experiments to verify its detection capability. First, the test of film thickness was conducted. Mark points were set every 50 mm on the steel plate as the detection target points, and a handheld instrument was used to collect the detection information as the standard value. Then, the experiment data were automatically detected and recorded after setting the detection mode and the advance distance of the robot. The specific experimental process and two groups of test data are shown in Figure 14.

The robot could move accurately and complete the corresponding detection process during the experiment, thus finally achieving the measurement and data recording of paint film thickness. A comparison of the two above-mentioned sets of data revealed that the thickness of the paint film automatically detected by the robot was 80 um, which was close to that detected manually. Allowable error bands (±0.5 um) were set with industrial testing requirements after consulting relevant testing manuals, and all the measured values of the marking points were found to be within the allowable error band. The maximum error was 0.3 um, appearing at the eighth marker, which also met the detection requirements.

**Figure 15:**
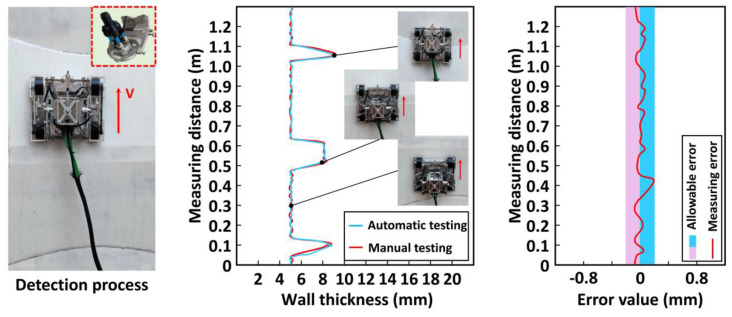
Measurement experiment of wall thickness.

An auxiliary device was necessary for the thickness detection of steel plates to provide the coupling liquid for the detection robot. The thickness of the steel plate was measured at the continuously changing splicing steel plate, and experiments of manual measurement and automatic continuous measurement were also conducted. The specific experimental process and two groups of test data are shown in Figure 15.

During the experiment, the robot could effectively complete the detection process and conduct automatic detection continuously. Similarly, allowable error bands (±0.2 mm) that met the requirements of industrial testing were set for wall thickness measurement. Figure 15 intuitively shows that the robot could continuously detect the thickness of the steel plate, and its thickness changed from 5 to 8 to 5 mm, which was similar to the manual measurement result and met the detection requirements. The two kinds of test data considerably fluctuated at the welding seam due to the influence of welding quality and position deviation of measurement points. Thus, the detection accuracy problem at the welding seam was temporarily disregarded. Stable data could be collected at other locations, and the test results met the test requirements. The maximum error of measurement was +0.2 mm, which was also within the required error range and met the detection requirements.

The above experiments revealed that the designed wall-climbing robot could adapt to the curved wall and move safely, smoothly, and flexibly on the wall. Vertical alignment detection could be realized by carrying ultrasonic and eddy current probes and by cooperating with the passive adaptation of the multi-DOF flexible detection mechanism, and the detection tasks of wall and paint film thicknesses could be effectively completed. The experimental results showed that the robot could complete the task of accurate wall stability detection and realize the automatic surface detection of petrochemical storage tanks in the degree of movement.

## 6. Discussion and Conclusions

A wall-climbing detection robot that can adapt to tanks with different radii of curvature was designed to address the increasing maintenance and testing requirements of petrochemical storage tanks. The robot realizes the non-destructive detection of the wall surface and its safe operation through human remote and automatic controls. Different from the traditional adsorption mechanism, the fan-shaped permanent magnet, which added a yoke to collect the magnetic induction line, is used as the excitation source in this robot. This adsorption mechanism reduces the weight of the magnetic wheel, improves the utilization rate of magnetic energy, and ensures reliable adsorption. In order to solve the problem that the existing adsorption devices are difficult to detach from a wall after completing detection, an innovative fast demagnetization mechanism was designed by using the lever principle. Considering that the traditional rigid moving mechanisms are difficult to adapt to different tank wall environments (different curvatures and various obstacles), a flexible adaptive moving mechanism with multi-DOFs was innovatively designed. The multi-DOFs flexible deformation of the moving mechanism can adapt to a wall surface, which ensures a close fit between magnetic wheels and the wall surface. A flexible detection mechanism that was designed on the basis of the hooke hinge mechanism can quickly change detection equipment to meet the technical requirements of film and wall thickness detections. Through the passive adaptation of a multi-DOFs hooke hinge mechanism, the detection probe can always be perpendicular to the center and close to the wall surface, thus meeting the requirements of accurate detection. Considering various working conditions, the minimum adsorption force and the optimal driving force range of straight line and turning motion were calculated by establishing the mechanical model, which ensured the flexible and stable movement of the robot on an arc facade. Finally, the precise coordination control of each component is performed by the wired control to complete the detection task while considering the limitation of the severe environment.

The wall-climbing detection robot was found to be able to move stably on a façade by conducting experiments on a facade with a thickness of 5 mm and a radius of 8 m, which verified the adsorption capacity of the magnetic wheel. The robot could complete large-radius and in-situ turning movements, which verified the wall surface adaptability of the robot’s moving mechanism. Through multisensor information fusion and multicomponent cooperation, the robot could complete the detection tasks of wall and paint film thickness detections by ultrasonic and eddy current sensors, respectively. The detection results also confirmed these findings. The experiment proved that the robot can complete the automatic wall detection task for petrochemical storage tanks.

## Figures and Tables

**Table 1:** Comparison of magnetic wheel characteristics before and after optimization.

Variate	Inner Radius $R_{in}$ (mm)	Angle of Magnet θ (°)	Yoke Iron Height H (mm)
Before optimization	25	70	6
After optimization	20	80	5

**Table 2:** Adsorption force of the magnetic wheel under different conditions.

Times	Horizontal	Vertical	Oblique
First time	121 N	124 N	120 N
Second time	119 N	122 N	123 N
Third time	123 N	129 N	123 N

**Table 3:** Parameters in the mechanical model.

Symbol	Comment	Symbol	Comment
$F_{fi}$	Friction of the robot	G	Weight force of the robot
$F_{Ni}$	Support force of the wheel	s	Safety parameter
$F_{Mag}$	Adsorption of the wheel	μ	Static friction coefficient
l	Length of robot	B	Width of robot
ω	Angular velocity of turning state	$h_{c}$	Centroid height of the robot

**Table 4:** Parameters in the mechanical model.

Symbol	Comment	Symbol	Comment
$V_{s}$	Velocity of the center of mass	$V_{l}$	Velocity of the left two wheels
$V_{r}$	Velocity of the right two wheels	$R_{w}$	Radius of magnetic wheel
R	Radius of gyration	α	Angular acceleration of robot
$T_{P}$	Moment of resistance of wheel	$T_{t}$	Motor output torque

**Table 5:** Keys technical parameters of the robot.

Items	Parameters
Weight	11 kg
Load capacity	9 kg
Maximum speed	10 m/min
Boundary dimension	400 × 400 × 300 mm
Communication mode	Wired (RS485)
Detection modes	Film/Wall thickness detection
